# Shiga Toxin Subtypes of Non-O157 *Escherichia coli* Serogroups Isolated from Cattle Feces

**DOI:** 10.3389/fcimb.2017.00121

**Published:** 2017-04-11

**Authors:** Pragathi B. Shridhar, Chris Siepker, Lance W. Noll, Xiaorong Shi, T. G. Nagaraja, Jianfa Bai

**Affiliations:** ^1^Department of Diagnostic Medicine and Pathobiology, Kansas State UniversityManhattan, KS, USA; ^2^Veterinary Diagnostic Laboratory, Kansas State UniversityManhattan, KS, USA

**Keywords:** shiga toxin, subtypes, non-O157 *E. coli*, cattle, human

## Abstract

Shiga toxin producing *Escherichia coli* (STEC) are important foodborne pathogens responsible for human illnesses. Cattle are a major reservoir that harbor the organism in the hindgut and shed in the feces. Shiga toxins (Stx) are the primary virulence factors associated with STEC illnesses. The two antigenically distinct Stx types, Stx1 and Stx2, encoded by *stx*1 and *stx*2 genes, share approximately 56% amino acid sequence identity. Genetic variants exist within Stx1 and Stx2 based on differences in amino acid composition and in cytotoxicity. The objective of our study was to identify the *stx* subtypes in strains of STEC serogroups, other than O157, isolated from cattle feces. Shiga toxin gene carrying *E. coli* strains (*n* = 192), spanning 27 serogroups originating from cattle (*n* = 170) and human (*n* = 22) sources, were utilized in the study. Shiga toxin genes were amplified by PCR, sequenced, and nucleotide sequences were translated into amino acid sequences using CLC main workbench software. Shiga toxin subtypes were identified based on the amino acid motifs that define each subtype. Shiga toxin genotypes were also identified at the nucleotide level by *in silico* restriction fragment length polymorphism (RFLP). Of the total 192 STEC strains, 93 (48.4%) were positive for *stx*1 only, 43 (22.4%) for *stx*2 only, and 56 (29.2%) for both *stx*1 and *stx*2. Among the 149 strains positive for *stx*1, 132 (88.6%) were *stx*1a and 17 (11.4%) were *stx*1c. Shiga toxin 1a was the most common subtype of *stx*1 among cattle (87.9%; 123/140) and human strains (100%; 9/9) of non-O157 serogroups. Of the total 99 strains positive for *stx*2, 79 were *stx*2a (79.8%), 11 (11.1%) were *stx*2c, 12 (12.1%) were *stx*2d. Of the 170 strains originating from cattle feces, 58 (34.1%) were *stx*2a subtype, 11 (6.5%) were *stx*2c subtype, and 11 were of subtype *stx*2d (6.5%). All but one of the human strains were positive for *stx*2a. Three strains of cattle origin were positive for both *stx*2a and *stx*2d. In conclusion, a number of non-O157 STEC serogroups harbored by cattle possess a wide variety of Shiga toxin subtypes, with *stx*1a and *stx*2a being the most predominant *stx* subtypes occurring individually or in combination. Cattle are a reservoir of a number of non-O157 STEC serogroups and information on the Shiga toxin subtypes is useful in assessing the potential risk as human pathogens.

## Introduction

Shiga toxin producing *Escherichia coli* (STEC) are major foodborne pathogens responsible for human illnesses, characterized by non-bloody to bloody diarrhea, sometimes leading to complications of hemolytic uremic syndrome (HUS), particularly in children (Gyles, 2007). *Escherichia coli* O157:H7 is the major serotype responsible for many of the STEC illness outbreaks in humans. However, there is increasing incidence of outbreaks associated with non-O157 STEC in recent years, particularly O26, O45, O103, O111, O121, and O145, referred to as top six non-O157 STEC. According to FoodNet sites, incidence of top six non-O157 STEC infections increased from 0.12 per 100,000 population in 2,000 to 0.95 per 100,000 population in 2010 (Gould et al., 2013). Non-O157 STEC associated illnesses range from cases of sporadic to major outbreaks, and clinically, from mild watery diarrhea to life threatening complications of HUS, similar to STEC O157 infections (Johnson et al., 2006). Cattle are a major reservoir of O157 and non-O157 STEC, which harbor the organisms in the hindgut and shed in the feces. Consumption of water, beef and fresh produce contaminated with cattle feces leads to human illnesses. In addition to O157 and the six top non-O157, cattle do harbor and shed in the feces a number of other serogroups of STEC (Bettelheim, 2007; Hussein, 2007).

Shiga toxins (Stx) are the major virulence factors of STEC. Shiga toxins (Stx) belong to the AB_5_ family of protein toxins, with an enzymatically active A moiety and a B moiety involved in binding to the host cell receptor. The A subunit is responsible for the cleavage of N-glycosidic bond in the 28 s rRNA of 60 s ribosomal subunit, which leads to cytotoxicity (Endo et al., 1988; Fraser et al., 1994). The two antigenically distinct Stx types, Stx1 and Stx2, encoded by *stx*1 and *stx*2 genes, share approximately 56% amino acid sequence similarity (Strockbine et al., 1986; Weinstein et al., 1988). Although Stx1 and Stx2 are structurally similar, they differ in cellular distribution and cytotoxicity. Shiga toxin 1 is located in the periplasmic space of the bacterial cell, whereas Stx2 is in the extracellular fraction (Shimizu et al., 2009). Basu et al. (2016) have shown that A_1_ subunit of Stx2 has a higher affinity for ribosomes and a higher catalytic activity compared to A_1_ subunit of Stx1 (Basu et al., 2016), which makes Stx2 more cytotoxic than Stx1. Shiga toxin 2 is reported to be 400 times more toxic than Stx1 in a murine infection model (Tesh et al., 1993). Shiga toxin 2 is more commonly associated with complications of HUS than Stx1 (Ethelberg et al., 2004; Brooks et al., 2005). Variants exist within *stx*1 (*stx*1a, *stx*1c, and *stx*1d) and *stx*2 (*stx*2a, *stx*2b, *stx*2c, *stx*2d, *stx*2e, *stx*2f, and *stx*2g) families based on differences in amino acid compositions and in the degree of cytotoxicity. The outcome of human illness associated with STEC strains has been shown to be influenced by Stx subtypes (Friedrich et al., 2002). Shiga toxin 2a and Stx2c are the major subtypes produced by *E. coli* O157:H7 strains associated with HUS in humans (Persson et al., 2007). Therefore, identifying the subtypes of (Stx) is important to assess the potential risk for human illnesses associated with STEC infections. Subtyping method based on restriction fragment length polymorphism of PCR products (PCR-RFLP) has been developed to identify *stx*1 (*stx*1a, *stx*1c, and *stx*1d) and *stx*2 (*stx*2, *stx*2v-ha, *stx*2v-hb, *stx*2g, *stx*2-NV206, and *stx*2-EC1586) genotypes at the nucleotide level based on their unique restriction patterns (Gobius et al., 2003; Beutin et al., 2007). However, the demerits of this method are the lack of consistency in the nomenclature, and misinterpretation of *stx* subtypes due to single nucleotide changes (Scheutz et al., 2012). Scheutz et al. (2012) standardized the Stx nomenclature by designating *stx*1 subtypes as *stx*1a, *stx*1c and *stx*1d, and *stx*2 subtypes as *stx*2a, *stx*2b, *stx*2c, *stx*2d, *stx*2e, *stx*2f, and *stx*2g based on amino acid sequence similarity (Scheutz et al., 2012). There is a paucity of data on Shiga toxin subtypes carried by non-O157 *E. coli* serogroups isolated from cattle feces in the United States. The objective of our study was to determine the subtypes of *stx*1 and *stx*2 in non-O157 *E. coli* serogroups isolated from cattle feces.

## Materials and methods

### Strains

Shiga toxin gene-positive *E. coli* strains (*n* = 192) spanning 27 non-O157 *E. coli* serogroups isolated from cattle feces (*n* = 170), and human clinical cases (*n* = 22), available in our culture collection, were used in the study. A majority of strains belonged to the top six non-O157 *E. coli* serogroups: O26 (*n* = 16), O45 (*n* = 4), O103 (*n* = 54), O111 (*n* = 21), O121 (*n* = 4), and O145 (*n* = 27). The other non-O157 *E. coli* serogroups included O6 (*n* = 2), O8 (*n* = 3), O15 (*n* = 1), O22 (*n* = 1), O38 (*n* = 2), O39 (*n* = 3), O74 (*n* = 3), O88 (*n* = 3), O91 (*n* = 2), O96 (*n* = 3), O104 (*n* = 18), O113 (*n* = 3), O116 (*n* = 3), O117 (*n* = 3), O130 (*n* = 4), O141 (*n* = 3), O146 (*n* = 1), O153 (*n* = 1), O163 (*n* = 2), O171 (*n* = 3), and O172 (*n* = 2). Cattle strains were isolated from feces, primarily from commercial feedlots (Renter et al., 2005; Noll et al., 2015; Shridhar et al., 2016; Cull et al., 2017). A few human clinical strains, obtained from Michigan State University (MSU) and the Kansas Department of Health and Environment (KDHE), were also included in the study. The strains, stored at −80° C on cryo beads (CryoCare™, Key Scientific Products, Round Rock, TX), were streaked onto blood agar plates (Remel, Lenexa, KS), colonies were suspended in 50 μl distilled water, boiled for 10 min, centrifuged at 9,300 × g for 5 min, and supernatant was used for PCR amplification and subsequent sequencing of the amplicons.

### Primers design

Nucleotide sequences of *stx*1 and *stx*2 of *E. coli* O157 and non-O157 *E. coli* were downloaded from NCBI Genbank. The sequences were aligned using CLC Main Workbench (CLC Bio, Cambridge, MA), and the conserved regions flanking the Shiga toxin genes were selected to design the oligonucleotide primers for amplification of *stx*1 and *stx*2 genes (Table 1). Sequences of *stx*2f subtype appeared to be more divergent from the other subtypes, therefore, a separate set of primers were designed for amplification (Table 1). Primers were obtained from Integrated DNA technologies (Coralville, Iowa).

**Table 1:** **Target genes, primers used and size of amplicons**.

**Target gene**	**Primers**	**Sequence**	**Amplicon size (bp)**	**Reference**
*stx*1	Forward	GCTCAAGGAGTATTGTGTAATATG	1,233	This study
	Reverse	TCGCTGAATCCCCYTC		
*stx*2	Forward	CGAATCCAGTACAACGC	1,390	This study
	Reverse	CCCACATACCACGAATC		
*stx*2f	Forward	CGTCATTCACTGGTTGG	766	This study
	Reverse	GCTGAGCACTTTGTAACA		

### PCR assay conditions

The *stx* genes were amplified by touchdown PCR method, where the annealing temperature of each cycle was lowered gradually to avoid amplification of non-specific sequences (Don et al., 1991). PCR was performed using Eppendorf Mastercycler (Eppendorf, Hamburg, Germany).

PCR amplification protocol for *stx*1 included an initial denaturation at 94°C for 5 min, 10 cycles of touch-down PCR (denature: 94°C for 30 s, annealing: 56–51°C (Δ-0.5°C) for 30 s; and extension: 72°C for 1 min 45 s) followed by 30 cycles of regular PCR (denature: 94°C for 30 s, annealing: 51°C for 30 s; and extension: 72°C for 1 min 45 s). PCR amplification protocol for *stx*2 included an initial denaturation at 94°C for 5 min, 10 cycles of touch-down PCR (denature: 94°C for 30 s, annealing: 47–44°C (Δ-0.3°C) for 30 s; and extension: 72°C for 1 min 45 s) followed by 30 cycles of regular PCR (denature: 94°C for 30 s, annealing: 44°C for 30 s; and extension: 72°C for 1 min 45 s). All PCR reagents were obtained from TaKaRa Bio USA, Inc. (CA).

### Sequencing and data analyses

Amplified PCR products were visualized using a Qiaxcel capillary electrophoresis system (Qiagen, Valencia, CA) and purified using a QIAquick PCR purification kit (Qiagen). The purified PCR products were measured by a spectrophotometer (NanoDrop-Thermo Scientific, Wilmington, DE) to assess the DNA concentration and purity. PCR products and primers were shipped to Genewiz, Inc., (South Plainfield, NJ) for nucleotide sequencing. The chromatogram data were visualized using the CLC Main Workbench software for further analysis. All sequences were individually analyzed for conflicts, and secondary peaks, and consensus sequences were produced. Nucleotide sequences were translated to amino acid sequences after removing intergenic sequences. Shiga toxin subtypes were determined based on the amino acid motifs that define each *stx* subtype (Scheutz et al., 2012).

### *In silico* restriction fragment length polymorphism (RFLP)

A subset of strains (*n* = 68) were subjected to *in silico* RFLP for identification of *stx*1 (*stx*1, *stx*1c, and *stx*1d) and *stx*2 (*stx*2, *stx*2v-ha, *stx*2v-hb, *stx*2-NV206, *stx*2g, and *stx*2-EC1586) genotypes. For *stx*1, a 391–392 bp fragment starting with GAYTATCATGGACAAGACTCYGTT and ending with TGACGATACYTTTACAGTTAAAGTGG was digested with *Rsa*I, *Fok*I, *and Nci*I enzymes using CLC Main Workbench software to identify the unique restriction sites for each *stx*1 genotype. For *stx*2, a 270 bp fragment starting with ATGAAGAAGATGTTTATG and ending with CAGTTTAATAATGACTGA was digested with *Hae*III, *Rsa*I, *Fok*I, and *Nci*I enzymes (Beutin et al., 2007) using CLC Main Workbench software. Restriction patterns of *stx*1 and *stx*2 sequences of the non-O157 STEC strains were compared to that of the reference sequences from the NCBI database to identify the *stx*1 and *stx*2 genotypes.

### Cloning and sequencing of PCR products

Three strains belonging to serogroups O96, O113, and O130 that revealed double peaks in the chromatograms of *stx*2 sequences were subjected to cloning. PCR products were cloned using TOPO® TA Cloning kits and protocols by Invitrogen-Life Technologies (Grand Island, NY). Up to 12 transformants were selected and grown in Luria Bertani broth (LB; Becton, Dickinson Co., Sparks, MD) containing carbenicillin for 2 h. Clones obtained from the LB broth were subjected to sequencing using flanking M13 forward and reverse primers. The sequences were analyzed as mentioned above to identify the Shiga toxin subtypes.

## Results

Of the total 192 non-O157 STEC strains (170 cattle and 22 human strains) belonging to 27 serogroups tested in the study, 93 (48.4%) were positive for *stx*1 only, 43 (22.4%) for *stx*2 only, and 56 (29.2%) for both *stx*1 and *stx*2. A total of 149 strains belonging to 23 serogroups were positive for *stx*1, and of those 132 (88.6%) were *stx*1a and 17 (11.4%) were *stx*1c (Table 2). Of the 140 *stx*1 positive strains originating from cattle feces, 123 (87.9%) were *stx*1a subtype, and 17 (12.1%) were *stx*1c. Shiga toxin 1a was the only *stx*1 subtype found in human STEC strains (9/9; 100%). The *stx*1a was also the most common subtype of *stx*1 identified in top six non-O157 *E. coli* serogroups (Table 2). None of the strains were positive for more than one *stx*1 subtype.

**Table 2:** **Shiga toxin subtype distribution in non-O157 Shiga toxin-producing *E. coli* (STEC) serogroups of cattle and human origin (*n* = 192)**.

**Serogroup**	**Cattle (*n* = 170)**					**Human (*n* = 22)**				
	***stx*1a**	***stx*1c**	***stx*2a**	***stx*2c**	***stx*2d**	***stx*1a**	***stx*1c**	***stx*2a**	***stx*2c**	***stx*2d**
**TOP NON-O157 STEC (*N* = 126)**										
O26 (*n* = 16)	9		3			2		4 (2^a^)		
O45 (*n* = 4)	4									
O103 (*n* = 54)	54				1 (1^b^)					
O111 (*n* = 21)	14		13 (13^a^)			6		7 (6^a^)		
O121 (*n* = 4)								4		
O145 (*n* = 27)	11		8 (1^a^)	4	1	1		3 (1^a^)		1
Total	92	0	24	4	2	9	0	18	0	1
**OTHER NON-O157 STEC (*N* = 66)**										
O6 (*n* = 2)	1		1 (1^a^)	1						
O8 (*n* = 3)	3		1 (1^a^)		2 (2^b^)					
O15 (*n* = 1)		1		1 (1^c^)						
O22 (*n* = 1)	1		1 (1^a^)							
O38 (*n* = 2)	1		2 (1^a^)							
O39 (*n* = 3)			3							
O74 (*n* = 3)	3		1 (1^a^)	2 (2^d^)						
O88 (*n* = 3)	3		3 (3^a^)							
O91 (*n* = 2)	1		2 (1^a^)							
O96 (*n* = 3)	2		3 (2^a^)		1 (1^e^)					
O104 (*n* = 18)		16						2		
O113 (*n* = 3)			2 (1^e^)		2					
O116 (*n* = 3)	3		3 (3^a^)							
O117 (*n* = 3)	2			1	2 (2^b^)					
O130 (*n* = 4)	3		2 (1^a^)		2 (1^b^,1^f^)			1		
O141 (*n* = 3)	3		3 (3^a^)							
O146 (*n* = 1)	1		1 (1^a^)							
O153 (*n* = 1)	1		1 (1^a^)							
O163 (*n* = 2)	2		2 (2^a^)							
O171 (*n* = 3)	1		1 (1^a^)	2						
O172 (*n* = 2)			2							
Total	31	17	34	7	9	0	0	3	0	0

a *Contains strains positive for both stx1a and stx2a*.

b *Contains strains positive for both stx1a and stx2d*.

c *Contains strains positive for both stx1c and stx2c*.

d *Contains strains positive for both stx1a and stx2c*.

e *Contains strains positive for both stx2a and stx2d*.

f *Contains strains positive for stx1a, stx2a and stx2d*.

*Numbers in the parenthesis indicate the presence of multiple stx subtypes*.

**Figure 1:**
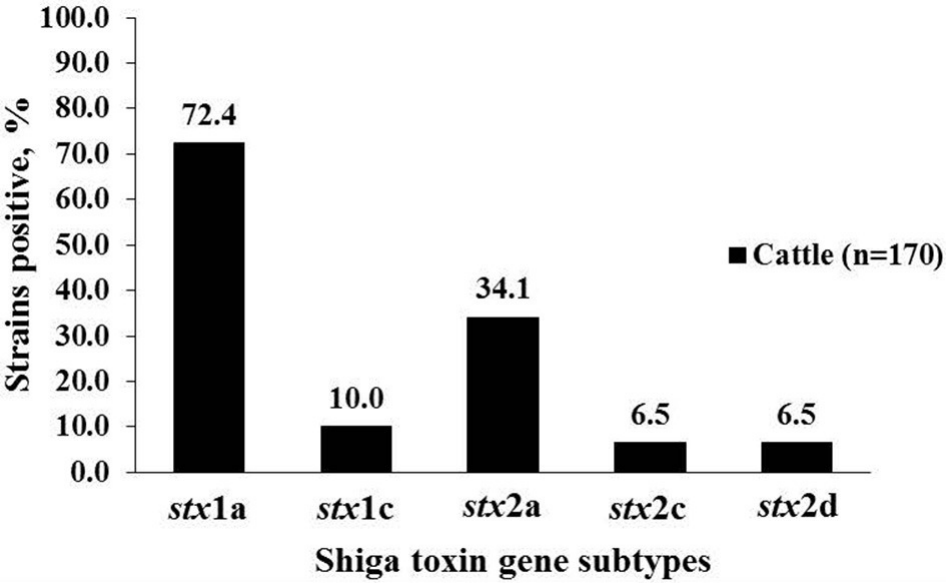
**Percentage of *stx*1 and *stx*2 subtypes in non-O157 Shiga toxin-producing *Escherichia coli* (STEC) strains isolated from cattle (*n* = 170)**.

A total of 99 strains belonging to 25 serogroups were positive for *stx*2, and of those 79 were *stx*2a (79.8%), 11 (11.1%) were *stx*2c, 12 (12.1%) were *stx*2d. Three *stx*2 positive-strains belonging to O96, O113 and O130 serogroups of cattle origin revealed double peaks in the chromatogram of *stx*2 sequences, suggesting the presence of more than one *stx*2 subtype in the same strain. Subsequent cloning and sequencing revealed that all three strains carried a combination of *stx*2a and *stx*2d. All human clinical strains (*n* = 22), except one, were of subtype *stx*2a. Only one strain belonging to serogroup O145 carried *stx*2d. All strains positive for *stx*2c were of bovine origin (Table 2, Figure 1). Shiga toxin subtypes 2e, 2f, and 2g were not detected in any of the strains tested. A majority of cattle strains (*n* = 170) possessed *stx*1a subtype (123/170; 72.4%) followed by *stx*2a (58/170; 34.1%; Figure 1). However, a majority of human strains carried *stx*2a (21/22; 95.5%) followed by *stx*1a (9/22; 40.9%). Shiga toxin 1a was most commonly found in combination with *stx*2a in strains (*n* = 46) belonging to 18 serogroups. Six strains belonging to O8, O103, O117, and O130 carried a combination of *stx*1a and *stx*2d. Two strains of serogroup O74 carried a combination of *stx*1a and *stx*2c and a strain of serogroup O15 was positive for a combination of *stx*1c and *stx*2c. A strain of serogroup O130 was positive for three subtypes, *stx*1a, *stx*2a, and *stx*2d (Table 2).

**Figure 2:**
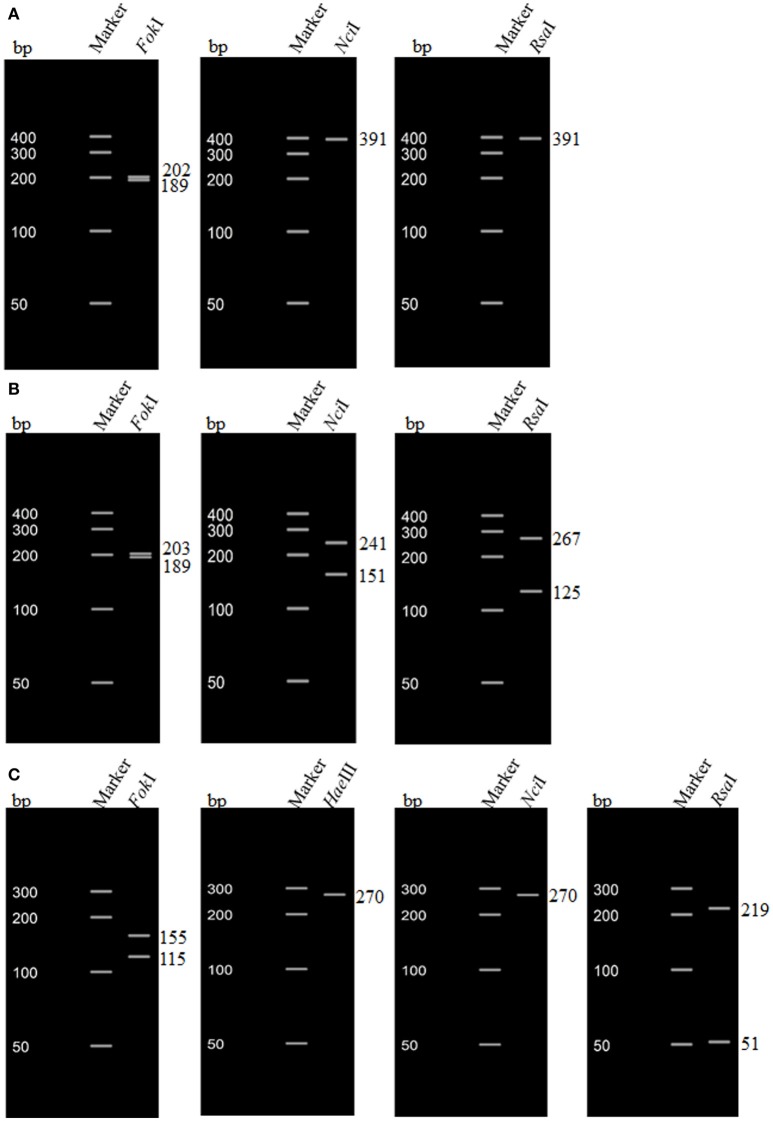
***In silico* restriction fragment length polymorphism (RFLP) of *stx*1 and *stx*2 genes of non-O157 Shiga toxin-producing *Escherichia coli* (STEC) strains. (A)** RFLP pattern of *stx*1a of a non-O157 STEC strain (O103 serogroup) isolated from cattle feces; **(B)** RFLP pattern of *stx*1c of a non-O157 STEC strain (O15 serogroup) isolated from cattle feces; **(C)** RFLP pattern of *stx*2 of a non-O157 STEC strain (O145 serogroup) isolated from cattle feces.

The sequences from a subset of strains (*n* = 68) were also analyzed by *in silico* RFLP. The sizes of fragments generated by restriction enzyme digestion are shown in Table 3. The two *stx*1 genotypes (*stx*1 and *stx*1c) determined by *in silico* RFLP corresponded to the two subtypes (*stx*1a and *stx*1c) determined based on the amino acid motifs. For *stx*2, genotypes, such as *stx*2, *stx*2-NV206, and *stx*2v-ha determined based on the restriction patterns corresponded to *stx*2a, *stx*2d, and *stx*2c subtypes, respectively, determined based on amino acid sequence. However, some strains positive for *stx*2v-hb gene (based on restriction patterns) corresponded to *stx*2c and some to *stx*2d subtype (based on amino acid sequence motifs). Restriction patterns of *stx*1 and *stx*2 genotypes determined by *in silico* RFLP are shown in Figure 2.

**Table 3:** **Restriction fragments of *stx*1 and *stx*2 genotypes based on *in silico* RFLP**.

**Shiga toxin subtypes**	**Restriction enzymes**			
	***Hae*III**	***Rsa*I**	***Fok*I**	***Nci*I**
	**Size(s) of restriction fragments (bp)**			
*stx*1	–	391	202, 189	391
*stx*1c	–	267, 125	203, 189	241, 151
*stx*2	270	219, 51	155, 115	270
*stx*2v-ha	143, 127	139, 80, 51	270	270
*stx*2v-hb	143, 127	219, 51	270	141, 129
*stx*2-NV206	270	219, 51	270	141, 129

## Discussion

Shiga toxins are the major virulence factors of STEC, which are responsible for foodborne illnesses, including life threatening complications of HUS in humans. Shiga toxins exist as several subtypes, which vary in their cytotoxicity, and therefore in the extent of their involvement in human illness (Friedrich et al., 2002; Persson et al., 2007; Fuller et al., 2011). Fuller et al. (2011) demonstrated that *stx*2a and *stx*2d subtypes were more potent than *stx*2b, *stx*2c, and *stx*1 based on *in vitro* (using primary human renal proximal tubule epithelial cells and Vero cells) and *in vivo* (using mice) experiments (Fuller et al., 2011). Karve and Weiss (2014) demonstrated stronger binding of *stx*2a, *stx*2c, and *stx*2d to a mixture of Gb3 and glycolipids when compared to *stx*1 and *stx*2b. Determining the Shiga toxin subtype carried by the STEC strains is important to estimate the risk of human illness associated with specific serotype or source of transmission. Studies have shown that STEC strains carrying *stx*2a and *stx*2c are most commonly associated with HUS in humans (Friedrich et al., 2002; Persson et al., 2007; Iyoda et al., 2014). Production of elastase activatable Stx2d subtype in STEC strains has been reported to be a predictor of severity of clinical illness (Bielaszewska et al., 2006). The cleavage of C-terminal amino acids of A_2_ peptide of Stx2d by elastase has been reported to increase the cytotoxicity of this Shiga toxin subtype (Kokai-Kun et al., 2000; Melton-Celsa et al., 2002). The Stx2e subtype is most commonly associated with porcine STEC, however, it is also associated with STEC from asymptomatic humans and fresh produce (Friedrich et al., 2002; Beutin et al., 2008; Feng and Reddy, 2013). Although pigeons are a primary reservoir of *stx*2f carrying *E. coli* strains, this subtype has also been isolated from human diarrheic patients (Schmidt et al., 2000; Prager et al., 2009). There are studies reporting the distribution of Shiga toxin subtypes in STEC strains isolated from humans and fresh produce (Friedrich et al., 2002; Feng and Reddy, 2013). There are very limited studies on the distribution of Shiga toxin subtypes in cattle, particularly of non-O157 serogroups, in the United States.

In this study, we identified Shiga toxin subtypes associated with 27 serogroups of non-O157 STEC strains of cattle (*n* = 170) and human (*n* = 22) origin based on the amino acid sequences deduced from nucleotide sequences. Additionally, we also identified *stx* genotypes by *in silico* RFLP, based on the restriction patterns obtained after *in silico* digestion of nucleotide sequences with specific restriction enzymes. The most common subtype of *stx*1 carried by non-O157 STEC strains of cattle and human origin was *stx*1a. A similar finding was also reported in a study on cattle and human STEC strains in Canada (Chui et al., 2015). Tostes et al. (2017) reported that *stx*1a and *stx*2a are the most common subtypes in both *E. coli* O157 and non-O157 *E. coli* strains of human and bovine origin in Alberta (Tostes et al., 2017). In the present study, a majority of the cattle STEC strains (21.8%; 37/170) and human STEC strains (40.9%; 9/22) carried a combination of *stx*1a and *stx*2a. A majority of *E. coli* O157:H7 strains isolated from outbreaks and sporadic cases in Canada carried a combination of *stx*1a and *stx*2a (Chui et al., 2015). In our study, 17 (10%) cattle STEC strains carried *stx*1c, while none of the human STEC strains carried this subtype. STEC strains carrying *stx*1c have been isolated from asymptomatic humans and patients with uncomplicated diarrhea (Friedrich et al., 2003). However, *stx*1c carrying *E. coli* O78 was isolated from a 2-week old boy suffering from bacteremia and HUS in Finland (Lienemann et al., 2012).

Shiga toxin subtype 2a was the most common *stx*2 subtype (41.1%; 79/192) found in cattle and human sources. It was the most common subtype detected in STEC strains isolated from patients suffering from HUS (Persson et al., 2007) and those isolated from fresh produce (Feng and Reddy, 2013). It was also the most common subtype of *stx*2 in STEC strains isolated from cattle in Australia (Brett et al., 2003) and France (Bertin et al., 2001). The second most frequent subtype of *stx*2 associated with STEC strains isolated from cattle was *stx*2c, however, none of the human STEC strains included in this study contained *stx*2c. The *stx*2c subtype has been reported in STEC strains isolated from human patients suffering from HUS (Persson et al., 2007). It was the third most common subtype (next to *stx*2d) associated with STEC strains isolated from fresh produce (Feng and Reddy, 2013). Tostes et al. (2017) have reported the distribution of *stx*1 and *stx*2 subtypes associated with *E. coli* O157 and non-O157 *E. coli* serogroups of bovine and human origin in Alberta, and found that the *stx*2c subtype was found only in *E. coli* O157 strains (Tostes et al., 2017). Shiga toxin 2c was the most common subtype associated with STEC O157 strains of cattle origin in Italy, however, none of the STEC O157 strains harbored *stx*2a (Bonardi et al., 2015). However, in our study, 6.5% of the non-O157 STEC strains isolated from cattle carried *stx*2c subtype. In the present study, only a small proportion (6.5%) of the bovine strains carried *stx*2d subtype, and only one human STEC strain carried *stx*2d. Tasara et al. (2008) have reported *stx*2d carrying STEC strains isolated from cattle and sheep (Tasara et al., 2008). Presence of *stx*2d carrying STEC strains in human patients was reported to be significantly associated with HUS (Bielaszewska et al., 2006). In our study, three cattle strains carried two different *stx*2 subtypes (*stx*2a and *stx*2d). STEC strains carrying more than one *stx*2 subtypes have been reported in previous studies (Persson et al., 2007; Scheutz et al., 2012). The significance of multiple *stx*2 subtypes with regard to virulence of STEC has not been studied yet. The presence of multiple *stx* subtypes in STEC is assumed to be the result of recombination of *stx* phages (Ashton, 2015).

We also identified Shiga toxin genotypes by *in silico* RFLP. Some strains positive for *stx*2v-hb gene (based on restriction patterns; Beutin et al., 2007) corresponded to *stx*2c and some to *stx*2d subtype (based on amino acid sequence motifs). Single nucleotide changes within the restriction sites could lead to misinterpretation of *stx* subtypes based on the restriction pattern (Scheutz et al., 2012). In conclusion, a number of non-O157 STEC serogroups isolated from cattle possessed a wide variety of Shiga toxin subtypes, with *stx*1a and *stx*2a being the most predominant *stx* subtypes occurring individually or in combination. Cattle harbor a number of non-O157 STEC serogroups and identification of the Shiga toxin subtypes is useful in assessing the potential risk to cause human illnesses.

## Author contributions

Conceived and designed the experiments: JB, TN; Performed the experiments: PS, CS, XS, LN; Contributed reagents/materials/analysis tools: JB, TN; Wrote the paper: PS, TN, JB.

## Funding

Research was supported by the Agriculture and Food Research Initiative Grant No. 2012-68003-30155 from the USDA National Institute of Food and Agriculture.

### Conflict of interest statement

The authors declare that the research was conducted in the absence of any commercial or financial relationships that could be construed as a potential conflict of interest.
